# Effects of high vs moderate-intensity intermittent training on functionality, resting heart rate and blood pressure of elderly women

**DOI:** 10.1186/s12967-020-02261-8

**Published:** 2020-02-17

**Authors:** Victor Silveira Coswig, Matheus Barbalho, Rodolfo Raiol, Fabrício Boscolo Del Vecchio, Rodrigo Ramirez-Campillo, Paulo Gentil

**Affiliations:** 1 College of Physical Education, Federal University of Pará, Castanhal, Pará Brazil; 2 College of Physical Education and Dance, Federal University of Goiás, Goiânia, Brazil; 3 Center for Biological and Health Sciences, University Center of the State of Pará, Belém, Pará Brazil; 4 Superior School of Physical Education, Federal University of Pelotas, Pelotas, Rio Grande do Sul Brazil; 5 Department of Physical Activity Sciences, Research Nucleus in Health Physical Activity, and Sport, Laboratory of Measurement and Assessment in Sport, Universidad de Los Lagos, Osorno, Chile; 6 FEFD-Faculdade de Educação Física e Dança, Universidade Federal de Goiás–UFG, Campus Samambaia, Avenida Esperança s/n, Campus Samambaia, Goiânia, Goiás CEP: 74.690-900 Brazil

**Keywords:** High-intensity interval training, Aerobic exercise, Old age home

## Abstract

**Background:**

The present study compared the effects of training and detraining periods of high-intensity interval training (HIIT), moderate-intensity interval training (MIIT) and moderate-intensity continuous training (MICT) on functional performance, body composition, resting blood pressure and heart rate in elderly women nursing home residents.

**Methods:**

Forty-six volunteers (age, 80.8 ± 5.2 y; body mass, 69.8 ± 5.2 kg, height, 164.2 ± 4.12 cm) were divided into groups that performed treadmill exercise twice-weekly HIIT (4 bouts of 4-min intervals at 85–95% of the maximal heart rate [HR_max_], interspersed by 4 min at 65% HR_max_), MIIT (4 bouts of 4 min intervals at 55–75% HR_max_, interspersed by 4 min at 45–50% HR_max_) and MICT (30-min at 55–75% HR_max_). Tests were performed before and after 8 weeks of training and 2 and 4 weeks of detraining. ANCOVA was used to analyze dependent variable changes.

**Results:**

After 8 weeks HIIT promoted greater reductions in body mass (HIIT = − 1.6 ± 0.1 kg; MICT = − 0.9 ± 0.1 kg; MIIT = − 0.9 ± 0.1 kg; p = 0.001), fat mass (HIIT = − 2.2 ± 0.1%; MICT = − 0.7 ± 0.1%; MIIT = − 1.2 ± 0.1%; p < 0.001) and resting heart rate (HIIT = − 7.3 ± 0.3%; MICT = − 3.6 ± 0.3%; MIIT = − 5.1 ± 0.3%; p < 0.001) and greater improvement in the chair stand test (HIIT = 3.4 ± 0.1 reps; MICT = 2.5 ± 0.1 reps; MIIT = 3.1 ± 0.1 reps; p < 0.001) when compared to MIIT and MICT. These improvements were sustained after 2 and 4 weeks of detraining only in the HIIT group.

**Conclusion:**

HIIT promoted greater benefits for body composition and functional performance than MICT and MIIT and also showed less pronounced effects of detraining. This suggests that the intensity of physical exercise is an important factor to consider when prescribing exercise to the elderly.

## Background

Aging is associated with a progressive decline in functionality [1]. Muscle mass and strength decrease ~ 12–14% per decade after the fourth decade of life [2]. In addition to aging, physical inactivity is associated with unfavorable changes in muscle structure and functional changes that are associated with decreased mobility and increased risk of falls, dynapenia, reduction in cardiorespiratory fitness and increased mortality [3, 4].

Physical exercise is recommended for older adults in order to increase aerobic capacity [5] and muscle strength [6]. Whilst the benefits of exercise programs for the elderly are well established [7], it is difficult to increase physical activity levels and exercise frequency in older populations [8], as well as, to promote long-term adherence [9]. To help solve these issues, exercise programs with low time commitment have been investigated [6, 10–12]. In this regard, high-intensity interval training (HIIT) might be particularly interesting.

HIIT consists of brief high-intensity efforts (> 85% HR_max_) interspersed with passive rest or low-intensity exercise periods [13]. HIIT is receiving increasing attention because of its benefits to cardiometabolic parameters in older people [14]. When compared to the moderate-intensity continuous training (MICT) that is traditionally indicated for the older people [7], HIIT produced similar or greater increases in aerobic capacity in healthy subjects and patients with heart diseases and metabolic disorders, as obesity and metabolic syndrome [15, 16], and also have similar adherence and enjoyment to MICT [17]. However, the feasibility of HIIT remains controversial because the metabolic and peripheral disturbances related to its intensity might be detrimental to adherence [18, 19]. Jimenéz-Pavón [20] suggested that the benefits obtained from HIIT in obesity, health parameters, and cardiovascular disease factors were not due to its intensity, but its intermittent characteristics. However, Gentil and Del Vecchio questioned this by suggesting that the benefits could actually be from the intensity itself [21]. This controversy underlines the complexity associated with interval training, which prevents the drawing of general conclusions [22].

Therefore, studies comparing different protocols are necessary to clarify this issue. If moderate-intensity interval training (MIIT) proves to be effective in elderly people, then it may be used as a preparatory/adaptive training prior to HIIT or even as a substitute for HIIT, as it would potentially reduce the intensity-related negative effects while maintaining the effectiveness of the exercise and increase adherence [20].

Detraining might be an important aspect to consider. In elderly patients with metabolic syndrome, blood pressure and waist circumference reductions persisted after 1 month of detraining from moderate-intensity interval aerobic protocols, but the gains in oxygen consumption and fat oxidation were completely reversed [23]. More importantly, the strength and functional losses of detraining are higher in the elderly than in younger people [24]. These accentuated losses might result in serious health consequences. A previous study in frail older people patients demonstrated that the interruption of exercise produced a substantial decrease in functionality with a high mortality rate [25]. Therefore, interruptions in training programs should be avoided to improve health status [5].

HIIT, MIIT, and MICT seem to have the potential to promote health-related benefits in elderly adults; however, their effects in training and detraining are not known and this knowledge would help professionals to better understand, manage, and prescribe aerobic-based exercises for the elderly. The present investigation compared the effects of training and detraining periods of HIIT, MIIT and MICT protocols on functional performance, body composition, resting blood pressure and heart rate of elderly women in an aged care facility. Our hypothesis is that HIIT would elicit higher increases in functional capacity and more pronounced decreases in body fatness when compared to the other protocols.

## Materials and methods

### Study overview

**Fig. 1:**
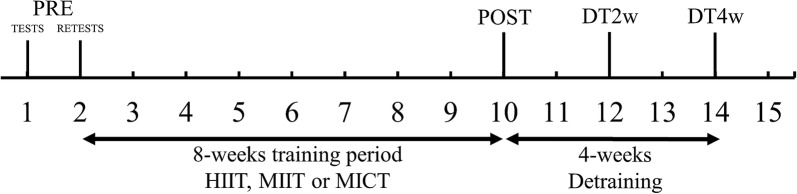
Experimental design

Forty-six participants were initially included and randomized in a blinded-counterbalanced manner into three groups based on their 6-min walking test results: HIIT (n = 15), MICT (n = 16) and MIIT (n = 15). Initially, participants were classified into terciles according to their 6-min walking test performance. Then, participants from each quartile were randomly assigned to one of the 3 groups. The first 2 weeks consisted of anthropometric evaluation and familiarization with the procedures, tests, and re-tests. During the familiarization sessions, the participants were instructed on proper exercise performance. Functional performance, a 6-min walking test, resting blood pressure and heart rate and bio-impedance assessments were performed prior and 5–7 days after 8 weeks of intervention. All tests were repeated two (DT2w) and four weeks (DT4w) after the post-intervention tests to assess detraining (Fig. 1). All tests were conducted by specialists blinded to group allocation. These tests were selected because of ease of application, popularity, reliability and clinical relevance [1].

### Participants

All participants were residents of the same nursing home and were there for social reasons or familiar decision. The following inclusion criteria were used: (i) free of any acute or chronic condition that would prevent the performance of tests and training protocols, (ii) 65 years of age or older, (iii) have not performed any type of aerobic training for at least 3 months prior to the intervention, (iv) be able to walk independently and (v) a minimum of 80% training attendance. In total, 83 women were nursing home residents, of which forty-six were eligible and volunteered themselves to participate in the study. There was no drop-out. The participants kept their normal routines, which consisted of performing the study’s determined physical activity program, social interaction activities and having their four meals a day. All participants were living in the same facility. The participants had some comorbidities that did not preclude to performed protocols and training, including hypertension (n = 16), type II diabetes mellitus (n = 14), Parkinson’s disease (n = 8), Alzheimer’s disease (n = 8). Participants were randomly allocated to each group and they maintained their regular treatment during the study period. All subjects were informed about the possible risks, benefits, and discomforts that the study could cause and signed an informed consent form. The relevant ethical committee approved the study under protocol No 68384217.1.0000.5172.

### Blood pressure and resting heart rate

Systolic and diastolic blood pressure (SBP and DBP) was measured after 5 min of seated rest (HBP-1100, OMRON, Brazil). Resting heart rate (RHR) was measured using a portable heart rate monitor (FT1, Polar, Finland). Blood pressure was measured using the auscultatory method following standard procedures [26].

### Nutritional control

All participants were full-time residents of the institution where the study was performed. All participants followed a meal plan that was divided into four meals per day during the entire intervention period, which did not change during the study period. Meals were scheduled at the same time every day (i.e., 7:00, 13:00, 17:00 and 20:00 h). Meal delivery was performed collectively, and subjects were supervised to ensure compliance. The following standard dietary plan was prescribed by a dietitian with the aim to provide an adequate energy balance: carbohydrate 4–6 g/kg/day, protein 0.8–1.0 g/kg/day, fat 0.6–0.8 g/kg/day, calcium 1000 mg/day, iron 8 mg/day, zinc 8 mg/day, magnesium 310 mg/day, phosphorus 700 mg/day, selenium 50 μg/day, vitamins D 15 μg/day, Vitamin A 650 μg/day, C 75 mg/day, Vitamin B12 2.4 μg/day, fibre 20 g/day, and water 2–3 l/day.

### Body composition

Lean mass and fat mass were assessed using a DSM-BIA (InBody 120, Biospace, Seoul, Korea), which is a multifrequency direct segmental bio-impedance apparatus with an 8-electrode tetrapolar system at 5, 50 and 500 kHz [27]. Participants were instructed to maintain a fasted state before the test. In the 24 h before the test, participants were instructed to avoid the ingestion of any substance or food that could influence body fluid content (i.e., caffeine, creatine, alcohol, tea, and foods with high sodium content) and to not exercise. All measures occurred at the same time of the day (between 6 and 8 a.m.). During the tests, the participants were bare feet and wearing light clothing. Two measurements were completed for each participant, 24–48 h apart, with an intraclass correlation coefficient (ICC) of 0.97–0.98.

### Functional performance

Functional performance was assessed by the following tests, performed in the order they are described. Each rest was separated by a minimum of 5 and a maximum of 10 min of passive rest.

### Gait velocity test

The walking velocity was evaluated across a 10 m distance. Participants were instructed to walk beginning on their dominant foot as fast as possible and proceed until the end of a marked path [28]. The time was measured using a handheld stopwatch. The test was performed twice, and the arithmetic mean from the two tests was used for scoring purposes. The results exhibited high reliability (ICC > 0.96, TEM = 0.05 m/s).

### 30-second chair stand test

This test was applied as previously described [1]. Briefly, the test involves counting the number of times the volunteer can rise to a full standing position from the seated position without using the arms. The results exhibited high reliability (ICC > 0.98, TEM = 0.28 rep).

### The six-minute walking test

This test was performed as previously described [29]. Participants were directed to walk along a 30 m track as fast as possible for 6 min. Verbal encouragement was provided during all tests, and the volunteers were allowed to rest during the test if necessary but were instructed to continue walking as soon as possible. The results exhibited high reliability (ICC > 0.94, TEM = 8.55 m).

### Training

The training was performed for 8 weeks, twice weekly, with ≥ 48 h of rest between sessions. The protocols were completed on a treadmill and intensity was prescribed on an individual basis and graded was used when necessary. The intensity was assessed and controlled using the maximal heart rate (HR_max_) and the formula proposed by Inbar et al. [30]. All groups underwent warm-up and cool-down periods of 5 min each at 65% HR_max_. Each volunteer used an HR monitor (Speedo Int Ltd, USA) during all sessions training to ensure compliance with the predetermined target HR zone. Velocity was increased by 0.1 to 0.5 km/h every time the HR was below the lower threshold at the end of the training session.

The three training protocols used in the study were:

High-intensity interval training (HIIT): 4 sets of 4-min intervals at 85–95% HR_max_ interspersed by a recovery period of 4 min at 65% HR_max_, for a total of 42 min of exercise per session, including warm-up and cool-down [31].

Moderate-intensity continuous training (MICT): 40 min of walking/jogging on the treadmill at 55–75% HR_max_, including warm-up and cool-down [31].

Moderate-intensity interval training (MIIT): 4 sets of 4-min intervals at 55–75% HR_max_ with a recovery period of 4 min at 45–50% HR_max_, for a total of 42 min of exercise per session, including warm-up and cool-down [32].

### Statistical analysis

The Shapiro–Wilk test was used to evaluate data normality. Descriptive data are presented as mean ± standard deviation (SD). Descriptive data were compared using a one-way analysis of variance (ANOVA). Body composition measures, functional performance, resting blood pressure and heart rate were compared within groups by one-way ANOVA for repeated measures. Bonferroni’s post hoc test was used when necessary. Analysis of covariance (ANCOVA) was used to compare absolute changes between groups using pre-test scores as a covariate and Bonferroni’s adjustment was applied. Absolute changes ± standard error (SE) are presented. Further, 95% confidence intervals (CIs) were examined for within-group changes. A significant within-group change occurred if the 95% CIs for changes did not cross zero.

Based on tests and re-tests, the standard error of measurement (SEM) was established for body mass, gait velocity, 30-s chair stand test, and 6-min walking test, as previously described [10]. Responsiveness was defined as changes that exceeded two times the SEM in favor of beneficial changes in the post-intervention period because this is considered the threshold for a true physiological adaptation beyond the expected results from technical and/or biological variability [10, 32]. The responsiveness threshold was set at 0.56 repetition for the 30-s chair stand test, 17.1 m for the 6-min walking test, 0.10 m/s for the gait velocity test and − 0.89 kg for body mass (TEM = 0.44 kg). Pearson’s Chi squared test was used to analyze the relationship between the distribution of responders and non-responders between groups. All tests were performed using SPSS 22.0 software, and statistical significance was set at p ≤ 0.05.

## Results

The baseline characteristics of the participants are presented in Table 1. No differences were found between groups for any variable. In addition, no adverse events occurred during the intervention period.

**Table 1:** Descriptive participant characteristics

	HIIT (n = 15)	MICT (n = 16)	MIIT (n = 15)
Age (years)	80.3 ± 5.8	81.2 ± 5.4	80.9 ± 4.6
Height (m)	1.64 ± 0.1	1.6 ± 0.1	1.6 ± 0.1
Body mass (kg)	69.3 ± 7.0	70.8 ± 3.9	69.2 ± 4.6
BMI (kg/m^2^)	25.6 ± 2.2	25.9 ± 1.9	26.1 ± 1.7
Fat mass (kg)	19.1 ± 5.4	19.4 ± 5.1	18.3 ± 4.3
Fat mass (%)	27.2 ± 5.7	27.2 ± 6.1	26.3 ± 5.3
Chair stand test (repetitions)	8.4 ± 1.4	8.5 ± 1.1	8.5 ± 0.8
6-min walking test (m)	406 ± 74.0	413 ± 58.3	403 ± 83.3
Gait velocity (m/s)	1.27 ± 0.11	1.27 ± 0.11	1.21 ± 0.11

*BMI* body mass index, *HIIT, MIIT, and MICT* high-intensity interval training, moderate-intensity interval training, and moderate-intensity continuous training groups, respectively

Table 2 shows body composition measures. All groups exhibited a significant reduction (p < 0.01) in body mass, fat mass and fat percentage after 8 weeks of training. However, after detraining these reductions were sustained only in the HIIT group, compared to baseline. For the MICT and MIIT groups, body mass, fat mass, and fat percentage were higher after 4 weeks of detraining compared to baseline. Fat-free mass did not change in any group.

**Table 2:** Anthropometric changes in elderly women after 8 weeks (post) of HIIT (n = 15), MIIT (n = 15), and MICT (n = 16) and 2 (DT2w) and 4 (DT4w) weeks of detraining

	Baseline	Post	DT2w	DT4w	ANOVA	
					F	p
Body mass (kg)						
HIIT	69.3 ± 6.9	67.7 ± 6.6^a^	68.2 ± 6.7^ab^	69.3 ± 6.7^bc^	61.7	< 0.001
MICT	70.8 ± 3.9	69.8 ± 3.7^a^	70.8 ± 3.7^b^	72.3 ± 3.6^abc^	219.9	< 0.001
MIIT	69.2 ± 4.6	68.2 ± 4.3^a^	69.0 ± 4.3^b^	70.0 ± 4.4^abc^	127.1	< 0.001
Fat mass (kg)						
HIIT	19.1 ± 5.3	17.1 ± 5.3^a^	17.8 ± 5.3^ab^	18.7 ± 5.4^abc^	187.6	< 0.001
MICT	19.4 ± 5.1	18.6 ± 4.8^a^	19.5 ± 4.8^b^	20.7 ± 5.0^abc^	142.2	< 0.001
MIIT	18.3 ± 4.3	17.3 ± 3.9^a^	18.0 ± 4.1^b^	19.0 ± 4.2^abc^	66.9	< 0.001
Fat mass (%)						
HIIT	27.2 ± 5.7	24.9 ± 5.8^a^	25.8 ± 5.8^ab^	26.6 ± 5.9^abc^	126.9	< 0.001
MICT	27.1 ± 6.1	26.4 ± 5.8^a^	27.4 ± 5.8^b^	28.5 ± 5.9^abc^	86.2	< 0.001
MIIT	26.3 ± 5.3	25.1 ± 5.1^a^	26.0 ± 5.1^b^	27.0 ± 5.1^abc^	66.2	< 0.001
Fat-free mass (kg)						
HIIT	29.4 ± 2.8	29.6 ± 2.7	29.3 ± 2.8	29.5 ± 2.9	0.068	0.976
MICT	30.1 ± 3.5	29.9 ± 3.6	29.7 ± 1.5	29.4 ± 1.6	0.262	0.852
MIIT	30.1 ± 3.2	30.1 ± 3.2	29.6 ± 1.8	30.1 ± 1.8	0.546	0.653

*DT* detraining, *HIIT, MIIT, and MICT* high-intensity interval training, moderate-intensity interval training, and moderate-intensity continuous training groups, respectively

^a^Different from baseline

^b^Different from POST

^c^Different from DT2w

The results of the functional tests are presented in Table 3. All groups exhibited a significant improvement in the chair stand test after training. However, the results were lower at DT4w than baseline for the MICT and MIIT groups. Only the HIIT protocol induced significant improvements in the 6-min walking test after 8 weeks of intervention. The HIIT group returned to baseline values at DT4w, and the MICT group exhibited lower values than the baseline. No group improved gait velocity and all groups exhibited a worse performance after detraining when compared to baseline.

**Table 3:** Functional performance test changes in elderly women after 8 weeks (post) of HIIT (n = 15), MIIT (n = 15), and MICT (n = 16) and 2 (DT2w) and 4 (DT4w) weeks of detraining

	Baseline	Post	DT2w	DT4w	ANOVA	
					F	p
Chair stand test (repetitions)						
HIIT	8.4 ± 1.4	11.8 ± 2.1^a^	10.2 ± 1.8^ab^	8.5 ± 1.9^bc^	105.1	< 0.001
MICT	8.5 ± 1.1	11.0 ± 1.6^a^	9.0 ± 1.3^ab^	6.4 ± 1.4^abc^	218.6	< 0.001
MIIT	8.5 ± 0.8	11.7 ± 1.1^a^	9.6 ± 0.9^ab^	7.4 ± 1.2^abc^	327.4	< 0.001
6-min walking test (m)						
HIIT	406 ± 73.5	454 ± 72.2^a^	431 ± 66.9^ab^	409 ± 75.1^bc^	123.5	< 0.001
MICT	413.1 ± 58.3	426.5 ± 67.6	393.1 ± 62.7^b^	365.0 ± 57.3^abc^	35.5	< 0.001
MIIT	403.3 ± 83.3	451.3 ± 83.5	427.3 ± 80.1	400.6 ± 76.2	1.657	0.191
Gait velocity (m/s)						
HIIT	1.27 ± 0.11	1.29 ± 0.07	1.13 ± 0.08^ab^	0.99 ± 0.07^abc^	90.5	< 0.001
MICT	1.27 ± 0.11	1.25 ± 0.1	1.12 ± 0.09^ab^	0.90 ± 0.13^abc^	20.9	< 0.001
MIIT	1.21 ± 0.11	1.24 ± 0.12	1.12 ± 0.08^ab^	0.98 ± 0.09^abc^	35.1	< 0.001

*DT* detraining, *HIIT, MIIT, and MICT* high-intensity interval training, moderate-intensity interval training, and moderate-intensity continuous training groups, respectively

^a^Different from baseline

^b^Different from POST

^c^Different from DT2w

Differences for SBP were found only after HIIT (Table 4). Post-training RHR values were lower than baseline and returned to baseline after DT4w for all groups.

**Table 4:** Resting blood pressure and heart rate changes in elderly women after 8 weeks (post) of HIIT (n = 15), MIIT (n = 15), and MICT (n = 16) and 2 (DT2w) and 4 (DT4w) weeks of detraining

	Baseline	Post	DT2w	DT4w	ANOVA	
					F	p
Systolic blood pressure (mmHg)						
HIIT	141.3 ± 8.3	138.0 ± 5.6^a^	138.7 ± 6.4^a^	140.7 ± 7.0	4.10	0.012
MICT	141.9 ± 13.3	141.3 ± 12.6	141.9 ± 13.3	141.9 ± 13.3	0.31	0.811
MIIT	140.7 ± 11.6	139.3 ± 9.6	140.0 ± 10.7	140.7 ± 11.6	1.63	0.195
Diastolic blood pressure (mmHg)						
HIIT	78.7 ± 7.4	77.3 ± 7.0	78.0 ± 6.8	78.7 ± 7.4	1.63	0.195
MICT	78.1 ± 6.6	77.5 ± 5.8	78.1 ± 6.6	78.1 ± 6.6	1.00	0.402
MIIT	77.3 ± 5.9	76.7 ± 4.9	77.3 ± 5.9	77.3 ± 5.9	1.00	0.333
Resting heart rate (bpm)						
HIIT	76.9 ± 5.7	69.5 ± 4.5^a^	72.5 ± 5.0^ab^	76.1 ± 5.2^bc^	199.40	< 0.001
MICT	76.2 ± 5.0	72.6 ± 4.8^a^	75.1 ± 4.5^b^	76.1 ± 4.8^b^	37.55	< 0.001
MIIT	76.7 ± 5.1	71.6 ± 5.2^a^	73.5 ± 4.7^ab^	76.5 ± 4.9^bc^	83.08	< 0.001

*DT* detraining, *HIIT, MIIT, and MICT* high-intensity interval training, moderate-intensity interval training, and moderate-intensity continuous training groups, respectively

^a^Different from baseline

^b^Different from POST

^c^Different from DT2w

The ANCOVA results are shown in Table 5. There were no significant differences between group for SBP (Baseline to post: F = 2.2, p = 0.114; post to DT2w: F = 0.02, p = 0.980; DT2w to DT4w: F = 1.3, 0.282) or DBP (Baseline to post: F = 0.2, p = 0.810; post to DT2w: F = 0.02, p = 0.976; DT2w to DT4w: F = 0.9, 0.407). Significantly greater reductions were found in RHR from baseline to post (F = 28.4; p < 0.001) after MIIT [− 5.1 ± 0.3 bpm (− 5.8 to − 4.3 bpm)] than MICT [− 3.6 ± 0.3 bpm (− 4.3 to − 2.9 bpm)], and HIIT [− 7.3 ± 0.3 bpm (− 8.0 to − 6.0 bpm)] produced greater reductions than MIIT and MICT.

**Table 5:** Changes in outcomes over the training and detraining periods (absolute changes ± standard deviation)

	HIIT (n = 15)	MICT (n = 16)	MIIT (n = 15)	ANCOVA	
	Change ± SD	Change ± SD	Change ± SD	F	p
Body mass (kg)					
Baseline-post	− 1.6 ± 0.1	− 0.9 ± 0.1^a^	− 0.9 ± 0.1^a^	8.2	0.001
Post–DT2w	0.5 ± 0.03	1.0 ± 0.03^a^	0.7 ± 0.03^ab^	71.8	< 0.001
DT2w–DT4w	1.1 ± 0.05	1.4 ± 0.04	1.1 ± 0.05	18.5	< 0.001
Fat mass (kg)					
Baseline-post	− 2.0 ± 0.07	− 0.7 ± 0.06^a^	− 1.0 ± 0.1^ab^	84.4	< 0.001
Post–DT2w	0.7 ± 0.07	0.9 ± 0.06	0.7 ± 0.07	3.0	0.058
DT2w–DT4w	0.8 ± 0.05	1.1 ± 0.05	0.9 ± 0.05	6.3	0.004
Fat mass (%)					
Baseline-post	− 2.2 ± 0.1	− 0.7 ± 0.1^a^	− 1.2 ± 0.1^ab^	42.6	< 0.001
Post–DT2w	0.8 ± 0.07	1.0 ± 0.07	0.9 ± 0.07	1.3	0.275
DT2w–DT4w	0.8 ± 0.07	1.0 ± 0.07	0.9 ± 0.07	1.3	0.275
Fat-free mass (kg)					
Baseline-Post	0.3 ± 0.04	− 0.2 ± 0.04^a^	0.1 ± 0.04^ab^	31.1	< 0.001
Post–DT2w	− 0.8 ± 0.5	0.1 ± 0.5	− 0.3 ± 0.5	0.6	0.532
DT2w–DT4w	0.2 ± 0.1	− 0.3 ± 0.1	0.4 ± 0.1^b^	5.2	0.009
Chair stand test (reps)					
Baseline-post	3.4 ± 0.07	2.5 ± 0.06^a^	3.1 ± 0.07^ab^	40.4	< 0.001
Post–DT2w	− 1.6 ± 0.1	− 2.0 ± 0.1	− 2.0 ± 0.1	3.3	0.045
DT2w–DT4w	− 1.7 ± 0.1	− 2.5 ± 0.1^a^	− 2.1 ± 0.1	7.6	0.001
6-min walking test (m)					
Baseline-post	47.5 ± 18.4	17.1 ± 17.9	45.1 ± 18.5	0.8	0.426
Post–DT2w	− 23.3 ± 2.3	− 33.2 ± 2.3^a^	− 24.1 ± 2.3^b^	5.4	0.008
DT2w–DT4w	− 21.9 ± 2.8	− 28.3 ± 2.7	− 26.4 ± 2.8	1.4	0.254
Gait velocity (m/s)					
Baseline-post	0.03 ± 0.02	− 0.01 ± 0.02	− 0.01 ± 0.02	1.1	0.332
Post–DT2w	− 0.16 ± 0.02	− 0.14 ± 0.02	− 0.08 ± 0.02	2.1	0.137
DT2w–DT4w	− 0.13 ± 0.02	− 0.20 ± 0.02	− 0.15 ± 0.02	2.7	0.076

*DT2w* detraining for 2 weeks, *DT4w* detraining for 4 week

^a^Different from HIIT

^b^Different from MICT

HIIT produced a greater reduction in body mass after training (F = 8.2; p = 0.001) and lower regain after detraining than MICT and MIIT (F = 71.8; p < 0.001). HIIT produced greater reductions in absolute and relative values of fat mass than MIIT and MICT, and MIIT produced greater reductions than MICT. HIIT produced greater improvements in the chair stand test after training compared to MIIT and MICT, and MIIT produced greater improvements than MICT. No between-group differences were found after training for the 6-min walking test, but the MICT group exhibited greater reductions after DT2w than HIIT or MIIT.

**Fig. 2:**
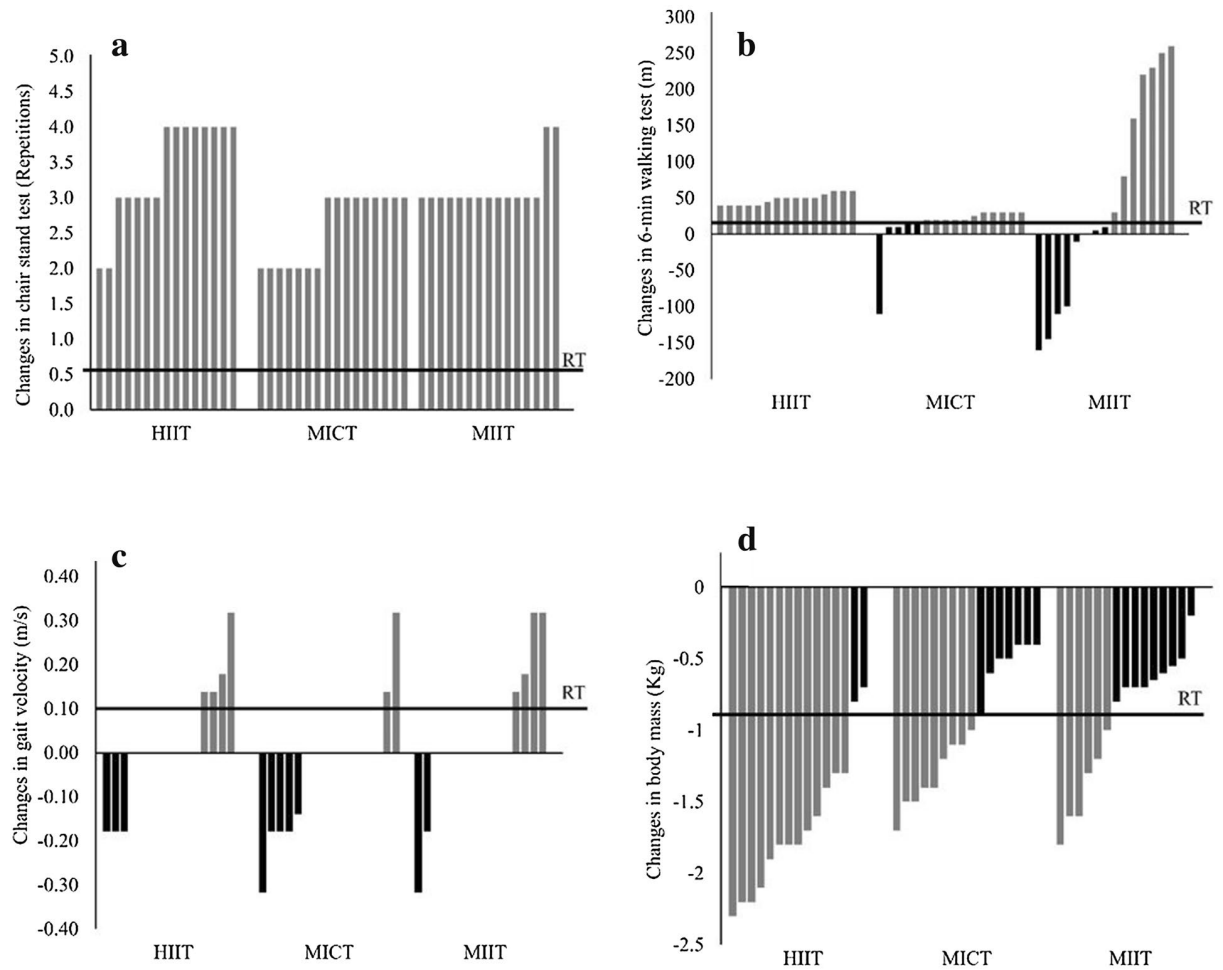
Individual absolute values and responsiveness thresholds of the chair stand test (**a**), the 6-min walking test (**b**), the gait velocity test (**c**) and body mass (**d**) in elderly women after 8 weeks of HIIT (n = 15), MIIT (n = 15), and MICT (n = 16). *HIIT, MIIT, and MICT* high-intensity interval training, moderate-intensity interval training, and moderate-intensity continuous training groups, respectively, *RT* responsiveness threshold

Considering responsiveness, there were no non-responders on the chair stand test in any group (Fig. 2a). For the 6-min walking test, there were no non-responders for the HIIT group. However, 38.5% and 61.5% of the volunteers were NR in the MICT and MIIT groups, respectively. Pearson’s Chi squared test revealed different distributions between MIIT and HIIT (χ^2^ = 14.1; p = 0.001; Fig. 2b). The gait velocity test demonstrated a high frequency of non-responders for HIIT (73.3%), MICT (87.5%) and MIIT (73.3%), with no significant differences in distribution between groups (χ^2^ = 1.31; p = 0.51; Fig. 2c). Body mass exhibited 11.8%, 35.3% and 52.9% NR for HIIT, MICT, and MIIT, respectively, with different proportions between HIIT and MIIT (χ^2^ = 7.4; p = 0.006, Fig. 2d).

## Discussion

This study compared the training and detraining effects of HIIT, MIIT, and MICT on body composition, functional performance, resting blood pressure and heart rate responses in elderly women living in a nursing home. The primary findings were: (i) Fat loss was higher after HIIT than after MICT and MIIT and returned to baseline after 4 weeks of detraining. (ii) HIIT and MIIT promoted higher improvements in the chair stand test than MICT, while only HIIT improved the 6-min walking test performance, in which the increases were higher than other protocols. No protocol increased gait velocity. (iii) After detraining, changes in functional performance were maintained only for HIIT, with the exception of gait velocity. (iv) All groups reduced RHR, and this reduction was greater for HIIT; after 4 weeks of detraining, the effects on RHR were completely reversed. (v) There were no non-responders in the chair stand test in any group, while for the 6-min walking test, there were no non-responders only in HIIT, and body mass responsiveness was higher in MIIT than in HIIT.

The results of body mass and fat loss were similar to previously reported using similar protocols in overweight women and metabolic syndrome patients [33, 34]. In a previous study, Zhang et al. [33] compared the effects of HIIT (4-min intervals at 85–95% HR_max_ and 3-min of active recovery at 50–60% HR_max_) and MICT (33 min at 60% HR_max_) in overweight women. Both protocols induced similar body mass changes (− 3.1% vs − 2.8%, for HIIT and MICT, respectively). However, HIIT induced greater loss of visceral (− 11.8 cm^2^ versus − 4.8 cm^2^, respectively) and subcutaneous fat (− 49.7 cm^2^ versus 25.4 cm^2^, respectively). Similar results were found for body fat (− 3.8%) and waist circumference (− 1.8%) in middle-aged patients with metabolic syndrome after 6 months of HIIT (4-min intervals at 85–95% HR_max_ and 3 min of active recovery at 50–60% HR_max_) [34].

Our study included MIIT, in which subjects executed a similar protocol to the HIIT group but at moderate intensity, in order to examine whether the benefits of HIIT were due to its intermittency or its intensity. Our findings suggest the MIIT promoted slightly better results in body composition than MICT, but statistically lower than HIIT, which suggests that intensity is an important factor to consider for this outcome, as previously suggested [21]. Indeed, despite the mean difference of only 0.7 kg could not sound meaningful, it is almost two times higher for the more intense protocol. However, the effects of intermittence cannot be neglected, since MIIT was superior to MICT in some aspects, which reinforces previous propositions of the potential benefits [20], whilst confirms that it might be inferior to HIIT [21].

The HIIT group maintained a reduction in fat mass even after 4 weeks of detraining, but the fat mass returned to baseline levels after 2 weeks of detraining in the MICT and MIIT groups, which seem to confirm the potential cardiometabolic risk of detraining [35]. Moreover, after 4 weeks of detraining, the MIIT and MICT groups continued to re-gain fat mass, reaching values greater than baseline. To explain this phenomenon, a compensatory effect in fat metabolism after the interruption of exercise should be considered, since previous studies suggest that there is an increase in lipogenesis after the cessation of exercise [36].

It should be highlighted that the different responses for each protocol could be explained by the unmatched volume proposed, as training methods were matched by total training duration. It may influence total energy expenditure and, consequently, energetic balance. However, despite our findings cannot address this issue, it might be possible that HIIT-induced changes in body composition are not restricted to calories burned, but also for biochemical and metabolic changes that improved fat oxidation capacity and raises basal energy expenditure [37, 38].

We found a significant reduction in systolic blood pressure, which was maintained after two weeks of detraining in the HIIT group. This result is consistent with previous results after six training sessions (15 × 1-min intervals at 90–95% HR_max_, separated by 1 min of active recovery at 70% HR_max_) [39]. Whilst a reduction in diastolic blood pressure was expected in response to MICT [40], this was not found, which may reflect the effects of aging in vasculature plasticity, leading to reduced responsiveness [40]. RHR decreased after all training protocols, which may be important for cardiovascular health [41]. After 2 weeks of detraining, this positive effect was maintained only after HIIT and MIIT.

Our findings revealed no differences in functional performance between groups. All groups improved performance in the chair stand test, but not gait velocity, and the 6-min walking test only improved after HIIT. These results contradict our hypothesis because we expected that the higher velocities achieved in the HIIT condition would promote greater increases in gait velocity due to specificity and following previous findings [42]. On the other hand, our data is not without support, since there is evidence in which the performance of HIIT or MICT promoted functional performance gains, but without differences between the types of training [43–45]. Considering the characteristics of the participants, these functional gains are very important, since they can decrease the risk of mortality, in which higher levels of functional capacity are related to lower risk of falls and mortality [46, 47]. According to our results, MICT showed a dramatic decline in almost all parameters after detraining. However, considering that old people might show functional and morphological declines over time, is not possible to be sure that the final results would have to be worst than performing no exercise.

Previous studies questioned the existence of non-responders to resistance training and HIIT in elderly people [10, 32] and emphasized the need to train at adequate training intensity [32, 48]. To the best of our knowledge, the present study is the first research to analyze responsiveness after MICT, MIIT, and HIIT. HIIT resulted in a lower overall prevalence of non-responders, but all participants exhibited benefits in at least one parameter when all tests performed were considered. These results are important because they reinforce the relevance of exercise prescriptions for elderly people and highlight the importance of continuity because many benefits were lost after short periods of detraining. Some parameters even worsened after detraining compared to baseline. Therefore, there should have no restriction in prescribing exercise for elderly people and it seems to be important to adopt strategies to stimulate long-term adherence and continuity.

The study has some strengths and limitations that should be acknowledged. First, we believe that dietary control is an important and difficult issue that we successfully addressed. As limitations, the differences in total work performed and the lack of a control group should be considered. However, we choose to not have a non-exercise group for ethical reasons, since older people are known to have a functional loss due to inactivity. Moreover, the differences in total work were inherent to the proposed protocols. The absence of peak performance measurements for training prescription (i.e. peak heart rate) and the use of prediction equations should be acknowledged; however, this procedure has high ecological validity and would be easier to implement in a real-life setting. Another important limitation is not having details of demographics, socioeconomic, alcohol consumption, and smoking status. Moreover, whilst it is suggested that HIIT protocols are safe and effective to improve functional capacity in older people, it is important to highlight the need for professional support for training implementation and monitoring. Future studies should include measures of balance and muscle strength tests, as they are important measures in frail elderly.

## Conclusion

Eight weeks of HIIT promoted greater benefits in body composition, resting blood pressure and heart rate and functional performance in elderly women when compared to MIIT and MICT protocols. In addition, the benefits of HIIT were sustained to a greater extent after detraining when compare to MIIT and MICT. Therefore, HIIT seems to be an efficient training strategy to promote morphological, physiological and functional benefits in elderly women.

## Data Availability

Not applicable.

## Abbreviations

HIIT: High-intensity interval training
MIIT: Moderate-intensity interval training
MICT: Moderate-intensity continuous training
HR_max_: Maximal heart rate
DT2w: Detraining for 2 weeks
DT4w: Detraining for 4 weeks
SBP: Systolic blood pressure
DBP: Diastolic blood pressure
BMI: Body mass index
ANOVA: Analysis of variance
ANCOVA: Analysis of covariance
CIs: Confidence intervals
SE: Stardard error
SEM: Stardard error of measurement
